# *CD4saurus Rex *&*HIVelociraptor *vs. development of clinically useful immunological markers: a Jurassic tale of frozen evolution

**DOI:** 10.1186/1479-5876-9-93

**Published:** 2011-06-16

**Authors:** Andrea De Maria, Andrea Cossarizza

**Affiliations:** 1 Centro di Eccellenza per la Ricerca Biomedica, Università di Genova, Genova, Italy; 2 Dipartimento Scienze della Salute (DISSAL), Università di Genova, Italy; 3 S.S. Infettivologia, Istituto Nazionale per la ricerca sul Cancro, Genova, Italy; 4 Dipartimento di Scienze Biomediche, Università di Modena e Reggio Emilia, Modena, Italy; 5 Departamento de Bioquímica y Biología Molecular, Universidad de Valencia, Valencia, Spain

**Keywords:** CD4+T cells, immune reconstitution, antiviral treatment, clinical trials

## Abstract

One of the most neglected areas of everyday clinical practice for HIV physicians is unexpectedly represented by CD4 T cell counts when used as an aid to clinical decisions. All who care for HIV patients believe that CD4+ T cell counts are a reliable method to evaluate a patient immune status. There is however a fatalistic acceptance that besides its general usefulness, CD4+ T cell counts have relevant clincal and immunological limits. Shortcomings of CD4 counts appear in certain clinical scenarios including identification of immunological nonresponders, subsequent development of cancer on antiretroviral teatment, failure on tretment simplification. Historical and recently described parameters might be better suited to advise management of patients at certain times during their disease history. Immunogenotypic parameters and innate immune parameters that define progression as well as immune parameters associated with immune recovery are available and have not been introduced into validation processes in larger trials. The scientific and clinical community needs an effort in stimulating clinical evolution of immunological tests beyond "*CD4saurus Rex" *introducing new parameters in the clinical arena after appropriate validation

## Introduction

Basic biomedical research is crucial for understanding pathogenetic mechanisms of diseases, as well as to develop new techniques drugs or concepts aimed at improving patient clinical care. Clinical implications of basic research are regularly reported in major journals in an effort to improve the transfer of benchwork into bedside clinical practices[1], and whenever promising steps in basic research fail to be introduced, this is underscored [2].

**Figure 1:**
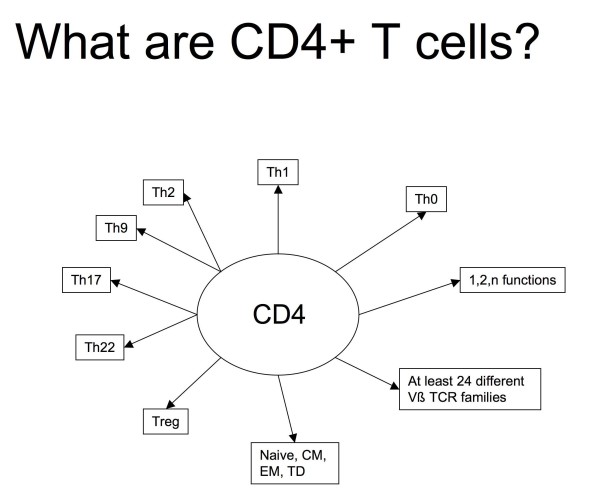
**Possible definitions of CD4+ T-cells based on current knowledge.** CD4+ cell counts only represent sums of individual subsets and do not reflect actual composition. Patients with equal CD4+ cell counts may reflect different proportional composition with possibly widely diverging functional immune characteristics.

After the identification of CD4+ T cells as the main target HIV replication, scientists focused on several important issues, including pathogenesis of mucosal infection[3-8], clearance of residual replication[9-13], evaluation of the involvement of innate immunity in disease progression[14-16], and identification of immunological correlates of protection for vaccine studies[17-20]. New cell subsets, receptors, cytokines, and signaling pathways were described [16,21-25], and widely available techniques for the *ex vivo *study of cells of the innate immune system (e.g. pDC, mDC, NK cells, NKTcells) or of Toll-like receptors were introduced. Models of HIV pathogenesis have been upgraded to account for and adjust to the new players and their functional characteristics. By "CD4" we now define at least 5 different CD4+ T-cell lineages, central and peripheral memory cells with variable effector functions (Figure 1). Evolution of our understanding of CD4+ T-cell type and function had however little impact on the Jurassik Park of clinical HIV care and antiretroviral trials. In the majority of cases, indeed, "immunology" is still represented by "quantitative determination of CD4+ T-cell numbers" alone to assess the immune status of routine patients attending HIV clinics.

### CD4saurus Rex: a relic from old times

For some reason, apparently unexplained factors prevented our clinical practice from evolving moving from discussing patients in terms of CD4+ T-cell counts alone to including additional specific tests. Clinical activity and knowledge has evolved rapidly in the same field leading to inclusion in everyday clinical life of DExa, PK/PD, ultrasensitive viral load, phenotypic and genotypic resistance evaluation. When it however comes to immunology, clinicians stick to a very successful but Jurassic test like CD4+ T-cell counts, which for the moment we could nickname "*CD4saurus Rex*". Unlike real Dinosaurs, CD4+ cell count and its clinical use has neither evolved/upgraded nor vanished each time a clinician is assessing a patient immune status before taking critical decisions with HIV patients.

There are few doubts that absolute CD4+ T cell counts have served, and still serve, as a very robust surrogate for progression to AIDS or death. They correlate generally well to the level of immune competence of patients with[26,27] or without[28] HIV infection, and also in children [29].

Proof of the robustness of this relic of immunological evolution is represented by its extensive use as a surrogate marker of immune competence to stratify or select patients in all recent trials evaluating or licensing the use of newer drug classes or ART regimens and in studies assessing the optimal time for ART initiation.

Even in the quest to identify additional risk factors for HIV-associated non-AIDS defining diseases, including nephropathy of cardiovascular disease, CD4+ T cell counts are the only immune parameter used to stratify patient cohorts[30,31].

### Shortcomings of CD4saurus Rex

The concept of CD4+ counts as clinical surrogate markers is still valid today and should continue to be used in routine patient follow-up. However, there are areas of clinical experience where it falls short of our needs.

Opportunistic infections may be sometimes observed in patients presenting with CD4+ T cell counts well above the critical range usually referred to for a given infection, as is the case for PML, TB[32-34], lymphoma[35] or Kaposi sarcoma patients who may unexpectedly present with relatively high CD4+ T cell numbers in the 300-400/μl range[36]. Conversely, at any given CD4+ cell count stratum, a fraction of patients have chances to develop PML, TB, lymphoma or KS, but CD4+ cell counts alone do not help us to further narrow down our attention on those who will actually develop disease. A surprisingly sustained incidence of HPV-associated pathology (e.g., cervical carcinoma, anal carcinoma) is observed even after successful ART with rising CD4+ cell counts[37,38]. Drug simplification to lopinavir/ritonavir monotherapy is effective at 96 weeks, but only in a subset of patients (47%) not identified by CD4+standards at baseline.[39].

These events are apparently unexplained to the clinician each time they are observed and are usually accepted as one would accept a rainy day. Other factors in the immune playground are likely to be involved, but "*CD4saururs Rex*" still grabs center stage, and we are missing part of the picture.

Treatment interruptions guided by the number of CD4+ T cells (CD4+GTI) have been lately discouraged by studies that showed significant risk of disease progression to AIDS or death or of cardiovascular events in patients selected on a narrow and low range of CD4+ T cells (i.e.350-200 CD4+/μl) [40]. Other studies however show that CD4+GTI is feasible and bears negligible/no risks for patients with different characteristics and considerably higher CD4+ cell counts (500-750/μl) [41,42]. Analysis of progressing patients on CD4+GTI shows that other phenotypic or functional immune variables beyond CD4+ cell counts (e.g.CD4+ nadir, NK cell phenotype[43]) may define the subset of patients for whom STI of CD4+GTI could be an option.

A similar issue is represented by immunological discordance on ART, which is observed in about 15-20% of drug-naïve patients starting ART[44]. Patients and physicians may develop anxiety over failure to recover CD4+ cells, over prophylaxis and over the risk of developing AIDS.

Thus, at the single patient level, which means our everyday life, we have to cope with an absolute number, without any other parameter that could help explain outliers, aid individualized management optimization or help to predict - or at least express a likelihood -whether a given patient will or will not develop an unwanted condition/disease course.

### What failed on the path of CD4+saurus Rex evolution, and which options are available

The reasons underlying this "frozen evolution" of immunological tests applied to HIV patients are multifaceted. Besides the outstanding robustness in terms of wide availability, standardization, and quality control of "*CD4saurus Rex*", there are few additional assays that raise some interest and that have been proposed as an additional qualitative or quantitative measure of the likelihood for a patient to develop a specific pattern of progression or of response to a given treatment.

**Figure 2:**
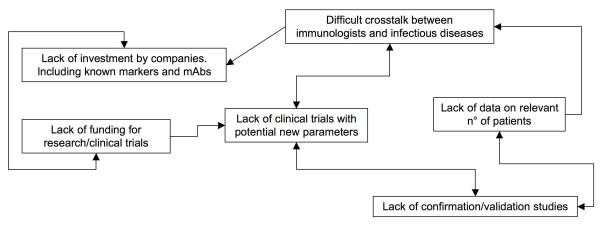
Why are we stuck with CD4+ alone to evaluate immune competence of HIV-1 patients?

The drive to improve basic understanding of HIV associated immune derangements could play a role in leaving little time and money to refine clinical use of acquired experience. Competition and a limited propensity of different researchers to integrate techniques developed elsewhere into clinical practice, also may play a role in the lack of translation of benchwork into bedside assets for patients. The misleading perception that budget restraints in immune evaluations are justified, particularly in the absence of wide integration of immunology centers, leaves us with large studies where CD4+ T-cells are the only immune measure, preventing inclusion of additional tests (Figure 2).

Alternative use of qualitative CD4+ T cell analysis, instead of CD4+ T cell count alone, has been proposed for other infectious diseases. It is well known that changes in the phenotype of CD4+ T cells occur in a large number of viral infections, and can be easily monitored. For example, atypical lymphocytes expressing CD4+/CD45RO+ may play the role of helper T cells in the development of the mononucleosis-like syndrome which is associated with Hepatitis-A infection[45]. During Epstein-Barr infection, a high number of CD4+Foxp3+ Treg cells can be localized in tonsils, which are the port of entry of the virus [46], and their role in inhibiting CD8+ T cell activity is under investigation. Recently, flow cytometry has also revealed its utility in providing informative patterns that can differentiate between infections of bacterial and viral origin. Indeed, these patterns have been obtained by combining the fractions of HLA-DR expressing T cell subpopulations with the level of CD40 on monocytes [47].

## Discussion

An improved approach to clinical development of immune measures should be designed and validated in the HIV arena. Embedding promising - or rather confirmed -immune parameters in new phase III/IV trials for ART or management optimization would provide a stimulus for the validation of additional clinical tools (Table 1). So far, no large study has tried to validate any of known potential markers which would add clinical information to CD4+ T-cell counts. These tests, including expression of CD38 or CD127, degree of T or NK cell activation (e.g.:HLA-DR, CD69 or Ki67), NCR expression by NK cells, or KIR:HLAtyping, could be used to flag different clinical options or outcomes at different times of the disease/treatment course (e.g.:immune-reconstitution, ART switch to monotherapy in selected patients or CD4+GTI, surveillance for unexpected opportunistic events including AIDS defining and non-AIDS defining neoplasms).

**Table 1:** List of useful or promising analyses, in addition to *CD4+saurus Rex *testing, so far unaccounted for in clinical trial validation but potentially relevant in every-day patient management and clinical decisions

What to test	Who and When	Possible use/interpretation	**Ref**.
**PBMC** **PD1^+^DR^+ ^Ki67^+^CD4^+^** **Ki67^+^PD-1^+^CD4^+Treg^** **Th17 cytokine profile**	Before cART in adv.naive and AIDS-presenter pts	Increase. Predict likelihood of IRIS. Diagnosis of IRIS (uppon symptoms	[59]

**Plasma TNF-a, IL-4, IL-17, VEGF, G-CSF, GM-CSF, CCL2(MCP-1)**	pt.with cryptococcal meningitis	Increase. Predicts high risk of IRIS	[60]

**PBMC** **CD56bright NK cells** **NKp30+CD56+, NKp46+CD56+ NK cells**	Before Voluntary or CD4+guided Treatment interruption	Increase. Advise against interruption for risk of rapid CD4 decrease when markers are increased	[43]

**PBMC** **CD4+/62L+/RA+** **CD8+/CD38+/DR+** **CD8+/62L+/RA+**	Wk16-24 of cART - Adolescents	Increase. Risk of Virological Failure after initial response	[61]

**PBMC** HLA-Bw4 (incl.HLA-B*57, HLA-B*27)	HIV infection, At first diagnosis	Presence. Defines lower risk of progression, chances of Elite Controlling, slow progression, lower VL	[62-65]

**PBMC** HLA-B*57 + KIR3DS1	Exposed uninfected partners, Any time	Presence. Decreased risk of infection upon HIV exposure	[66-68]

**PBMC** HLA-B*57 + KIR3DL1high	Exposed uninfected partners, Any time	Presence. Decreased risk of infection upon HIV exposure	[69]

HLA-B*57	HIV-Infected, Before cART start	Presence. Defines adverse reaction to Abacavir	[70]

**CCR5-∂32, CCR2-64I**	At diagnosis.	Presence. Slower disease progression, lower VL	[71]
	
	Before cART	Less time to undetectable VL, decreased risk of AIDS	[72,73]

**CD127+CD4+ T cells**	Before cART	Decrease. Immunological non-response to cART	[49,51,74]

**CD127+CD4+ and/or CD127+CD8+ T cells**	Before Voluntary or CD4+guided treatment interruption	Increase. Directly correlated with the length of treatment interruption	[75]

In industrialized countries, most laboratories are equipped with flow cytometers that are able to analyze routinely multiple (at least 4, up to 8) fluorescent cellular markers. More sophisticated approaches based on polychromatic flow cytometry have allowed to identify a relevant heterogeneity within the CD4+ T cell compartment [48]. Additional information on the immunological status of a given patient can be easily obtained, and should be customized on patient needs. Testing would not be required on a regular "routine" basis, but rather could be applied just before a "strategic management decision", to estimate the likelihood of a given patient or patient group to have different clinical outcomes. This would contain costs and provide optimal use of a dedicated test. For example, concerning management of a drug naïve patient, one of the main questions would be whether the patient will become an immunological non-responder. Thus, tests for assessing the capacity of producing new T-cells, in terms of thymic functionality (such as the amount of TREC+cells, IL-7 plasma levels, expression of CD127)[49-51] could be introduced. Similarly, KIR:HLA carriage and IL-28 Bpolymorphisms condition significantly treatment response during HCV infection[52-54] and has implications also on disease course in coinfected patients and possibly bear on response to ART[55-58]. Concerning an advanced patient failing a drug regimen, markers of CD8+ T cell activation (such as CD38, CD95 or MHC class II), differentiation (CD45RA, CCR7 or CD62L), survival (CD127) and of CD4 activation and differentiation could be crucial (Table 1). Similar considerations could be applied to successfully treated patients with suppressed VL and recovered CD4+ T cell count, who would candidate for simplification regimens (or for possible drug vacation on the basis of NK activating/inhibitory receptor phenotype[43] (Table 1). Last but not least, age-related immunological changes in a huge number of parameters, including the subpopulations of CD4+ T cells, have to be considered when "normal" levels of a biomarker are studied, considering that the aging of HIV+ patients is an emerging problem of relevant importance (50).

## Conclusion

In conclusion, time has come to introduce complementary customized parameters, in addition to CD4+ cell counts, in the clinical care of HIV-infected patients in order to provide additional immuno-virological stratification criteria. This may be achieved by specifically investing on appropriate validation/standardization strategies in clinical trials (extending in range from ART initiation to ART optimization) using available parameters

## Competing interests

The authors declare that they have no competing interests.

## Authors' contributions

ADM conceived the design of the commentary, discussed and wrote the manuscript. AC participated in the design of the commentary, discussed and wrote the manuscript. All authors read and approved the final manuscript
